# Synthesis of a Functionalized Benzofuran as a Synthon for Salvianolic Acid C Analogues as Potential LDL Antioxidants

**DOI:** 10.3390/molecules20058654

**Published:** 2015-05-14

**Authors:** Gabriela López-Frías, Alejandro A. Camacho-Dávila, David Chávez-Flores, Gerardo Zaragoza-Galán, Víctor H. Ramos-Sánchez

**Affiliations:** Facultad de Ciencias Químicas, Universidad Autónoma de Chihuahua, Circuito Universitario, Campus Universitario #2, Apartado Postal 669, Chihuahua, Chih. 31125, Mexico; E-Mails: gaby_ca23@hotmail.com (G.L.-F.); dchavezf@uach.mx (D.C.-F.); ger210582@yahoo.com.mx (G.Z.-G.); vramos@uach.mx (V.H.R.-S.)

**Keywords:** benzofuran, antioxidant, palladium

## Abstract

A palladium mediated synthesis of a common synthon for the syntheses of antioxidant analogues of naturally occurring salvianolic acids is presented. The synthetic route may be used to obtain analogues with a balanced lipophilicity/hydrophilicity which may result in potentially interesting LDL antioxidants for the prevention of cardiovascular diseases.

## 1. Introduction

Oxidative damage of low density lipoprotein (LDL) plays a key role in the pathogenesis of several disorders, especially cardiovascular diseases (CVD), atherosclerosis and hypercholesterolemia among many others [1]. Oxidative stress may result in an impairment of glucose uptake in type 2 diabetes [2]. It is a fact that lipid oxidation is associated with platelet activation therefore resulting in an increase on the risk of CVD such as thrombosis and stroke [3].

In recent years, the intake of naturally occurring antioxidants has been associated with a benefit for the prevention of several conditions such as cancer, ageing, CVD and others [4,5]. These observations suggest that fruits and vegetables may have some protective effect against oxidative damage in the body [6,7,8]. However, as previously reported, the mechanisms of absorption, distribution and metabolism may have an effect on the effectiveness of these natural antioxidants [9,10,11].

**Figure 1:**
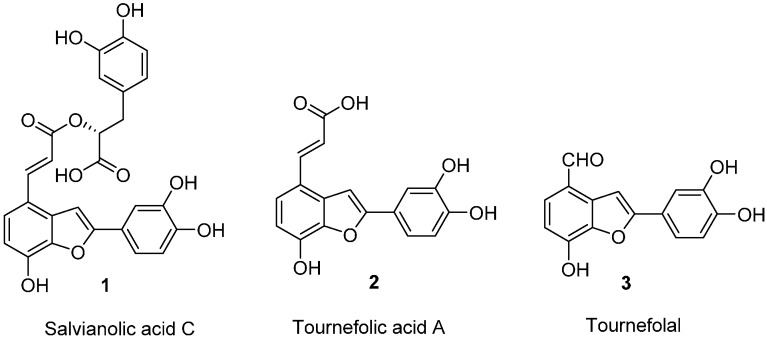
Structures of natural benzofuran antioxidants isolated from *Salvi*a species.

For many years, traditional Chinese medicine has been using different extracts and concoctions of native plants for the treatment of different conditions [12,13]. Among these plants, the Chinese plant danshen (*Salvia milthiorrhiza*) and the Taiwanese plant *Tournefortia sarmentosa* Lam. have been used as detoxicants, anti-inflammatory and have also been used as an aid to promote blood circulation, and in the treatment of other causes such as stroke, atherosclerosis and thrombosis [14,15]. From the aqueous extracts of these plants several compounds have been isolated (Figure 1), including salvianolic acid C (**1**) and some novel compounds such as tournefolic acid A (**2**) and tournefolal (**3**) [16]. All of which possess in common a polyphenolic structure and a benzofuran ring system [17,18]. All of the isolated compounds showed an important effect in preventing Cu^2+^-induced LDL oxidation, indicating that these polyphenolic compounds could prevent LDL oxidation by the scavenging of free radicals [19].

Some previous reports on pharmacokinetic studies have shown that water soluble compounds isolated from *S. miltiorhiza* possess strong antioxidant and lipid protective activities [20]. It is reported that salivianolic acids exert a protective effect by scavenging oxygen free radicals [20,21]. One of the problems associated with these compounds is their poor bio-availability due to their high hydrophilicity [22] resulting from the presence of the catechol moiety, and thus being metabolized to other mostly methylated analogues from which the biological activity is unknown [23]. Therefore, considering the important activity of these derivatives as potential therapeutical agents for the treatment of coronary and circulatory diseases, the development of novel analogues, possessing structures which can be modified through the attachment of different functionalities to modify their lipohilicity/hydrophilicity, is important in order to obtain analogues for biological assays [24,25].

One of the main factors for the antioxidant activity of these compounds is the presence of the catechol function. This is present in many naturally occurring antioxidant compounds such as flavonoids which are of great importance due to the health associated benefits obtained from their consumption [26,27,28,29,30]. However, bioavailability depends on a balance between lipophilicity and hydrophilicity, making this factor essential for the biological activity of this type of compounds [22,31]. Thus, finding a balance between these two factors could be a way to develop analogues with better absorption and improved biological activity [31,32]. Additionally, the benzofuran moiety is also present in biologically active natural products possessing antiviral, antitumor, antioxidant and antifungal activities, among others [33].

In connection with another project, we needed to synthesize some analogues of the reported tournefolic acid A **2** and tournefolal **3** containing a site where some other functionality could be installed to modify their lipophilic properties while maintaining a catechol structure intact. Hence, in this paper we present an approach to a functionalized benzofuran scaffold which can be further functionalized by the attachment of different functional groups while maintaining the catechol from which analogues of either salvianolic acid C or tournefolic acid A could be obtained in order to evaluate the potential biological activity of the obtained derivatives. 

**Scheme 1:**
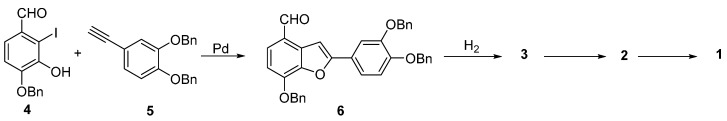
Synthetic route to salvianolic acid derivatives.

Previously, coumpounds **1**–have been obtained using the tandem palladium-copper mediated cross-coupling/cyclization of 2-iodo-3-hydroxy-4-benzyloxybenzaldehyde **4** and the benzylic protected alkynyl catechol **5** followed by deprotection of benzofuran **6** by reductive elimination of the benzyl protecting groups to afford tournefolal (**3**) from which **1** and **2** were obtained as shown in Scheme 1 [34]. 

**Scheme 2:**
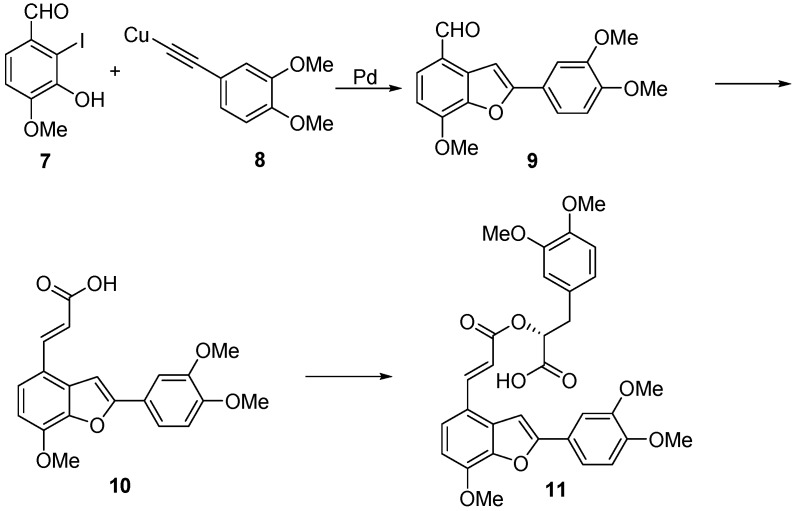
Synthetic route to pentamethylsalvianolic acid C **11**.

In another report [25], the synthesis of a pentamethyltournefolic acid A **10** was obtained through the same reaction between a functionalized o-iodophenol **7** and a functionalized copper phenylacetylide **8** to yield the trimethoxytournefolal **9** from which **11** was obtained (Scheme 2). Further functionalization of pentamethyltournefolic acid A **10** furnished the pentamethylsalvianolic acid C **11**. Attempts to remove the methyl groups by different strategies resulted in a complete failure to furnish any of the desired salvianolic acid C. This result has been observed with similar substrates containing a benzofuran ring system [35].

As we were interested in the synthesis of analogues of salvianiolic acid C (**1**), tournefolic acid A (**2**) and tournefolal (**3**) from a common intermediate, which could be functionalized at the phenolic position *para* to the cinnamic acid chain of **2** in order to modify the lipophilicity, while maintaining the catechol structure in ring C, we were faced with the problem of finding a properly functionalized derivative which could be easily deprotected under mild conditions and without disturbing any other functionality installed in the molecule in order to access all required derivatives from a single common intermediate.

**Scheme 3:**
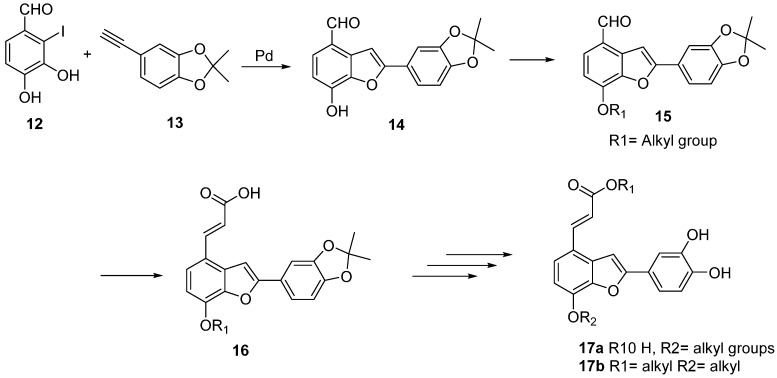
Proposed synthetic route to salvianolic acid analogues.

Initially, we considered the use of the palladium-mediated coupling between *o*-halophenols and alkynes in a similar way as the previously descried synthesis of **1** and **2** [35]. This methodology has been used successfully in the synthesis of diversely functionalized benzofurans [36,37]. Hence, palladium-mediated cross coupling between ethynylcatechol **13** with *o*-haloaldehyde **12** (Scheme 3) could furnish the corresponding protected tournefolal ketal **14** which is a scaffold ready to attach further functionalities into the free phenolic group by alkylation of the phenol group of **14** with different alkyl halides to produce functionalized tournefolal ketal **15**. 

Attachment of the propenoic acid chain to aldehyde **15** will produce alkylated tournefolic acid A ketal **16**, which can be further manipulated at the carboxyl function to install the required functionality to produce analogues **17** of either **1** or **2**. This way, several analogues of tournefolic acid A or salvianolic acid C can be obtained to be evaluated for their biological activity. In this paper we report the progress made in this approach.

## 2. Results and Discussion

**Scheme 4:**
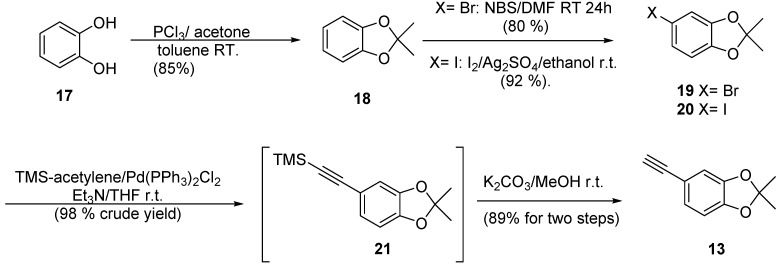
Synthetic route to alkyne precursor **13**.

As a starting material and as a source of ring C of the benzofuran scaffold, we selected the inexpensive and readily available catechol (**17**) and isovanillin (**22**) as the source of A ring. Initially (Scheme 4), protection of the phenolic groups in catechol **17** was achieved by reaction between **17** and acetone in the presence of phosphorus trichloride to afford the cyclic ketal **18** in 85% yield [38]. With ketal **18** readily available, halogenation with NBS in DMF afforded the corresponding bromide **19** in 80% yield [39,40]. Sonogashira coupling of **19** with trimethylsilylacetylene resulted in a poor yield of the coupling product **21** attributed tothe electron richness of the ring [41]. Consequently, the change of bromine for iodine was required in order to improve the yield in this step. Reaction of ketal **18** with iodine in ethanol in the presence of silver sulfate afforded the corresponding iodo compound **21** in 92% yield [42]. Sonogashira coupling of **21** with TMS-acetylene [43] and removal of silicon under standard conditions (K_2_CO_3_/methanol) furnished the desired alkyne **13** in 89% yield. 

**Scheme 5:**
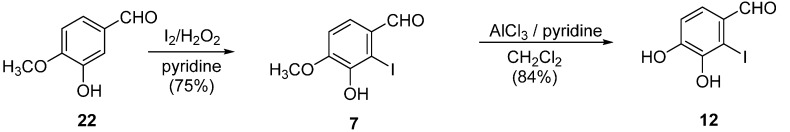
Synthetic route to iodophenol precursor **12**.

The fragment **12** corresponding to ring A was obtained starting from isovanillin **22** as previously reported [44]. Compound **12** was obtained in 65% overall yield (Scheme 5). 

**Scheme 6:**
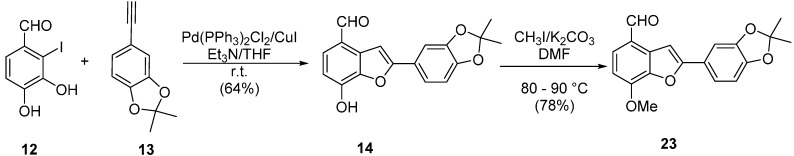
Scheme for Pd-catalyzed coupling reaction to benzofuran ring system.

Having obtained the necessary precursors, the coupling-cyclization reaction was performed (Scheme 6). Thus, treatment of a solution of iodide **12** in DMF with alkyne **13** in the presence of copper (I) iodide (4 mol %), Pd(PPh_3_)_2_Cl_2_ (2 mol %) and triethylamine (3 equivalents) at 65–70 °C for 12 h afforded a mixture of products from which the desired tournefolal ketal **14** was detected in the crude reaction TLC by their intense blue fluorescence under short UV-light. Purification of the reaction mixture by flash column chromatography resulted in the isolation of pure tournefolal ketal **14** in 64% yield. No attempts to optimize this reaction by changing the catalyst or their ratio were performed. 

To evaluate the reactivity of the phenol group with alkylating agents, the reaction of **14** with iodomethane in potassium carbonate to afford the methoxybenzofuran aldehyde **23** was performed, resulting in a 78% yield, thus demonstrating that this position can be readily functionalized. Deprotection of **14** by stirring with neat trifluoroacetic acid at room temperature afforded after purification the previously reported tournefolal (**3**) in 70% yield.

**Scheme 7:**
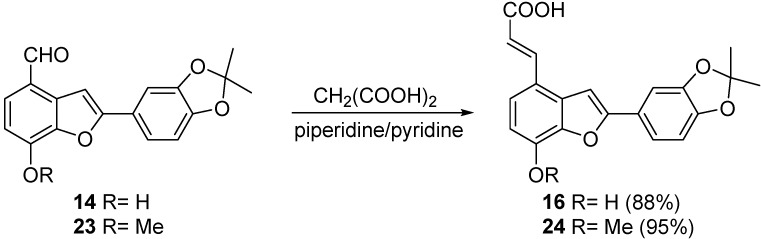
Functionalization route to salvianolic acid C synthon.

Formation of the propenoic acid chain was accomplished by reaction of protected hydroxytournefolal **14** or methoxytournefolal **23** with excess of malonic acid in pyridine containing a catalytic amount of piperidine at room temperature, thus affording the protected acids **16** and **24** in good yields (Scheme 7). Acid **16** is a convenient starting material for the syntheses of either derivatives of salvianolic acid C or tournefolic acid A which can be functionalized at the free phenolic position. 

## 3. Experimental Section 

### 3.1. General Methods 

Anhydrous THF was distilled over sodium benzophenone ketyl under N_2_; all other solvents and reagents were used from commercial sources without further purification. The silica gel (230–400 mesh, NATLAND, Durham, NC, USA) was used for column chromatography. ^1^H-NMR spectra were recorded at 500 MHz and ^13^C-NMR spectra were recorded at 125 MHz on a Varian V NMR System instrument (Varian Instruments, Palo Alto, CA, USA) Chemical shifts (δ) are given in ppm with reference to either to TMS (0.00) or the corresponding solvent signals [^1^H-NMR: CDCl_3_ (7.27), CD_3_SOCD_3_ (2.5). ^13^C-NMR: CDCl_3_ (77.0), CD_3_SOCD_3_ (39.52); CD_3_COCH_3_ (29.84). HRMS was recorded on a GC-Mate II GC-MS (Jeol, Tokyo, Japan). Low resolution MS was obtained on a Turbo Mass Gold mass spectrometer (Perkin-Elmer, Waltham, MA, USA).

### 3.2. Synthesis

*5-Iodo-2,2-dimethylbenzo[d][1,3]dioxole* (**20**). 2,2-Dimethylbenzo[d][1,3]dioxole [38] (**18**, 10 g, 66.58 mmol), Ag_2_SO_4_ (20.75 g, 66.58 mmol) and I_2_ (16.91 g, 66.58 mmol) were stirred for 2 h in anhydrous ethanol (250 mL) at room temperature. After this time, the iodine color disappeared completely and the mixture was filtered to eliminate the precipitated salt. Ethanol was removed on a rotary evaporator and the residue was then dissolved in CH_2_Cl_2_ (100 mL) and the organic phase was washed with a 20% Na_2_S_2_O_3_ solution. The organic phase was separated, dried over Na_2_SO_4_, filtered and concentrated under reduced pressure to yield the practically pure iodoketal **20** as a yellow oil (16.91 g, 92%). This was used in the next step without further purification. ^1^H-NMR (CDCl_3_) δ: 7.08 (dd, *J* = 8.1, 1.8 Hz, 1H); 7.02 (d, *J* = 1.8 Hz, 1H); 6.50 (d, *J* = 8.1 Hz, 1H); 1.65 (s, 6H). ^13^C-NMR (CDCl_3_) δ: 148.56, 147.63, 130.00, 118.80, 117.48, 110.41, 81.63, 25.93. MS (CI): *m*/*z* 276 (M+, 100%), 261, 149. 93, 63.

*((2,2-Dimethylbenzo[d][1,3]dioxol-5-yl)ethynyl)trimethylsilane* (**21**). Into a 100 mL r.b. flask equipped with a magnetic stirring bar were placed iodoketal **20** (9.63 g, 34.88 mmol), CuI (132 mg, 2.0 mol %) and Pd(PPh_3_)_2_Cl_2_ (979 mg, 4 mol %). The flask was stoppered with a septum and purged with Ar. Distilled THF (50 mL), Et_3_N (14 mL, 100 mmol) were successively added trough a syringe and the mixture was stirred for 15 min. After this time, ethynyltrimethylsilane (7.39 mL, 52 mmol, 1.5 equiv) was slowly added through a syringe, after a few minutes the mixture spontaneously warmed and the addition was continued maintaining the temperature below 35 °C. After the addition, the mixture was stirred 4 h and then diluted with ethyl acetate (100 mL). The mixture was filtered through Celite containing a layer of activated charcoal. The filtrate was concentrated under reduced pressure yielding a dark brown syrup. This was used in the next step without further purification. (Crude yield: 8.25 g, 96%). ^1^H-NMR (CDCl_3_): δ 6.96 (dd, *J* = 8.0, 1.6 Hz, 1H); 6.82 (d, *J* = 1.6 Hz, 1H); 6.64 (d, *J* = 8.0 Hz, 1H); 1.66 (s, 6H); 0.22 (s, 9H). MS (CI): *m*/*z* 246 (M+), 232, 191, 108 (100%), 43.

*5-Ethynyl-2,2-dimethylbenzo[d][1,3]dioxole* (**13**). To a stirred solution of TMS alkynylketal **21** 5 g (20.3 mmol) in anhydrous methanol (50 mL), solid K_2_CO_3_ (5.52 g, 40 mmol) was added and the mixture was vigorously stirred during 4 h. After this time, the mixture was filtered over Celite and the Celite washed with methanol (20 mL). Removal of methanol under vacuum yielded a brown oil which was purified by column chromatography eluting with hexane/EtOAc (9/1) to yield the pure alkyne ketal **13** (3.15 g, 89%). ^1^H-NMR (CDCl_3_): δ 6.99 (dd, *J* = 8.0, 1.6 Hz, 1H); 6.86 (d, *J* = 1.6 Hz, 1H); 6.67 (dd, *J* = 8.0, 0.5 Hz, 1H); 2.97 (s, 1H); 1.67 (s, 6H). ^13^C-NMR (CDCl_3_): δ 148.20, 147.23, 126.42, 118.7, 114.63, 111.91, 108.34, 89.83, 75.41, 25.89). MS (CI): *m/z* 174 (M+), 159 (100%), 135, 88 62, 50, 43. HRMS: Calculated for C_11_H_10_O_2_: 174.0681, found: 174.0681.

*2-(2,2-Dimethylbenzo[d][1,3]dioxol-5-yl)-7-hydroxybenzofuran-4-carbaldehyde* (**14**). To a two necked 100 mL r.b. flask equipped with a magnetic stirring bar was added successively 210 mg Pd(PPh_3_)_2_Cl_2_(210 mg, 3 mol %), CuI 76 mg (4 mol %), of 3,4-dihydroxy-2-iodobenzaldehyde [44] (**12**, 2.6 g 10 mmol) and DMF (35 mL). After this, the flask was purged several times with argon and triethylamine (5 mL) was added via syringe. The mixture was heated in an oil bath at 80–90 °C and then a solution of alkyne **13** (1.456 g 8.36 mmol, 1.2 equiv) in DMF (8 mL) was added via syringe during 30 min and after this, heating was continued for 20 h. TLC (SiO_2_, hexane/EtOAC 7/3) showed the presence of furan **14** as an intense blue fluorescence under short wave UV light. After the heating period, the volatiles were removed under reduced pressure and the residue adsorbed into silica gel which was directly applied to the chromatographic column packed with silica gel. Elution with hexane/EtOAc in a gradient (7/3, 6/4, 5/5) afforded pure benzofuran **14** as a dark brown powder (1.98 g, 64%). ^1^H-NMR (CDCl_3_): δ 10.03 (s, 1H); 7.63 (s, 1H); 7.61 (d, *J* = 8.2 Hz, 1H); 7.40 (dd, *J* = 8.1, 1.7 Hz, 1H); 7.25 (d, *J* = 1.8 Hz, 1H); 6.91 (d, *J* = 8.1 Hz, 1H); 6.79 (d, *J* = 8.1 Hz, 1H); 1.71 (s, 6H). ^13^C-NMR (CD_3_SOCD_3_) δ: 190.91, 159.52, 148.77, 148.10, 145.85, 139.22, 131.99, 130.57, 123.11, 122.56, 119.45, 118.84, 110.31, 108.62, 105.60, 101.02, 25.87. HRMS Calculated for C_18_H_14_O_5_: 310.0841, found: 310.0840.

*2-(3,4-Dihydroxyphenyl)-7-hydroxybenzofuran-4-carbaldehyde (Tournefolal)* **3**. Benzofuran aldehyde **14** (100 mg, 0.322 mmol) was added to a cooled solution of neat trifluoroacetic acid (2 mL). The mixture was stirred at room temperature during 16 h. The trifluoroacetic acid was removed *in vacuo* and the residue was purified by column chromatography on Sephadex LH20 eluting with methanol. Compound 3 was obtained as a greenish powder (61 mg, 70%). The spectral data agreed with the reported values [19,34]. ^1^H-NMR (CD_3_SOCD_3_): δ 9.98 (s, 1H); 7.67 (d, *J* = 8.2 Hz, 1H), 7.55 (s, 1H); 7.34 (d, *J* = 2.1 Hz, 1H); 7.30 (dd, *J* = 8.2, 2.1 Hz, 1H); 6.88 (d, *J* = 9 Hz, 1H); 6.86 (d, *J* = 8.2 Hz, 1H). ^13^C (CD_3_SOCH_3_): δ 190.42, 158.83, 147.91, 147.26, 145.72, 142.49, 131.58, 130.37, 120.77, 120.58, 117.27, 116.21, 112.38, 110.20, 99.16.

*(E)-3-(2-(2,2-dimethylbenzo[d][1,3]dioxol-5-yl)-7-hydroxybenzofuran-4-yl)acrylic acid* (**16**). Benzofuran aldehyde **14** (310 mg, 1 mmol) was dissolved in pyridine (5 mL) and then malonic acid (1 g, 9.56 mmol) and a drop of piperidine was added. The mixture was stirred at room temperature during 5 days and then heated in an oil bath at 85–90 °C for 1 h. The volatiles were removed under reduced pressure and the dark brown residue was purified by column chromatography eluting with a gradient of hexane/acetone (7/3, 6/4, 5:5) to afford acid **16** as a golden solid strongly fluorescent under short wave UV light (150 mg, 88%). ^1^H-NMR (CDCl_3_): δ 7.92 (d, *J* = 16.1 Hz, 1H); 7.63 (s, 1H); 7.57 (dd, *J* = 8.1, 1.8 Hz, 1H); 7.50 (d, *J* = 1.7 Hz, 1H); 7.47 (dd, *J* = 8.3, 0.6 Hz, 1H); 6.91 (dd, *J* = 8.2, 0.4 Hz, 1H); 6.86 (d, *J* = 8.3 Hz, 1H); 6.52 (d, *J* = 16.0 Hz, 1H); 1.71 (s, 6H).^13^C CD_3_COCD_3_) 168.30, 158.11, 149.34, 149.03, 145.01, 143.48, 131.79, 126.21, 124.59, 119.90, 119.81, 119.63, 116.41, 111.87, 109.37, 106.15, 100.33, 25.89 HRMS: Calculated for C_20_H_16_O_6_: 352.0947, found: 352.0934.

*2-(2,2-Dimethylbenzo[d][1,3]dioxol-5-yl)-7-methoxybenzofuran-4-carbaldehyde* (**23**). To a solution of aldehyde **14** (750 mg, 2.41 mmol) in DMF (5 mL) finely powdered K_2_CO_3_ (750 mg, 5.42 mmol) was added. The mixture was stirred and to this, iodomethane (0.5 mL, 8.03 mmol) was added in one portion. The mixture was heated in an oil bath at 70–80 °C for 24 h. The reaction mixture was cooled and diluted with chloroform (50 mL). The mixture was diluted with water and the phases separated. The organic phase was washed with water (6 × 25 mL) and dried over Na_2_SO_4_. Solvent removal at reduced pressure and column chromatography (SiO_2_) of the residue eluting with hexane/ethyl acetate 4/1 afforded pure **23** as a dark yellow solid (610 mg, 78%). ^1^H-NMR (CDCl_3_): δ 10.04 (s, 1H); 7.64 (d, *J* = 8.3 Hz, 1H); 7.62 (s, 1H); 7.44 (dd, *J* = 8.1, 1.8 Hz, 1H); 7.29 (d, *J* = 1.7 Hz, 1H); 6.86 (d, *J* = 8.3 Hz, 1H); 6.80 (d, *J* = 8.1 Hz, 1H); 4.13 (s, 3H); 1.71 (s, 6H).^13^C-NMR (CDCl_3_): δ 190.74, 159.28, 149.59, 148.62, 148.04, 143.71, 131.0, 130.33, 123.3, 122.72, 119.45, 118.7, 108.55, 105.84, 105.68, 100.48, 56.44, 25.88. HRMS: Calculated for C_19_H_16_O_5_: 324.0998, found: 324.0993.

*(E)-3-(2-(2,2-dimethylbenzo[d][1,3]dioxol-5-yl)-7-methoxybenzofuran-4-yl)acrylic acid* (**24**). Aldehyde **23** (500 mg, 1.54 mmol) was dissolved in pyridine (5 mL) and then malonic acid (1 g, 9.56 mmol) and a drop of piperidine was added. The mixture was heated in an oil bath at 85–90 °C for 4 h. The mixture was poured into a mixture of ice (50 g) containing concentrated HCl (5 mL). The precipitated yellow solid was recovered by filtration and washed with water several times. The acid was dried at 50 °C in vacuum and then washed with dichloromethane. This way, pure **24** was obtained as a yellow solid (536 mg, 95%). ^1^H-NMR (CDCl_3_): δ 7.82 (d, *J* = 16.1 Hz, 1H); 7.74 (s, 1H); 7.56 (d, *J* = 8.4 Hz, 1H); 7.50 (d, *J* = 1.8 Hz, 1H); 7.48 (dd, *J* = 8.1, 1.8 Hz, 1H); 6.98 (d, *J* = 4.9 Hz, 1H); 6.96 (d, *J* = 4.5 Hz, 1H); 6.56 (d, *J* = 16.1 Hz, 1H); 4.02 (s, 3H); 1.69 (s, 6H). ^13^C-NMR (CD_3_SOCD_3_): δ 167.99, 156.62, 147.86, 147.6, 146.18, 142.81, 141.56, 129.33, 125.43, 123.07, 119.52, 118.84, 118.82, 117.37, 108.65, 107.29, 105.25, 99.97, 56.10, 25.54. HRMS: Calculated for C_21_H_18_O_6_: 366.1103, found: 366.1110.

## 4. Conclusions 

In summary, the synthesis of the natural benzofuran tournefolal (**3**) and its ketal protected derivative **14**, as well as ketal protected tornefolic acid A **16** have been synthesized to provide a convenient access to precursors of analogues of either salvianolic acid C (**1**) or tournefolic acid A (**2**). Further functionalization of aldehyde **14** with different alkylating agents could be realized in a straightforward manner as demonstrated by methylation with iodomethane to access diverse analogues to evaluate the effect of these chains in their biological action. Also, the aldehyde function can be transformed into other functionalities, thus increasing range of analogue syntheses. Synthetic studies of different alkylated derivatives and biological evaluation of these compounds are currently underway and will be reported in due course.

## Supplementary Materials

Supplementary materials can be accessed at: http://www.mdpi.com/1420-3049/20/05/8654/s1.
