# Supplementary material for: Synthesis of a Functionalized Benzofuran as a Synthon for Salvianolic Acid C Analogues as Potential LDL Antioxidants
Source: Molecules. 2015 May 14;20(5):8654–65. doi: 10.3390/molecules20058654 (PMC6272376; doi:10.3390/molecules20058654)
Supplement: Supplementary file 1 [file molecules-20-08654-s001.pdf]

Supplementary Materials

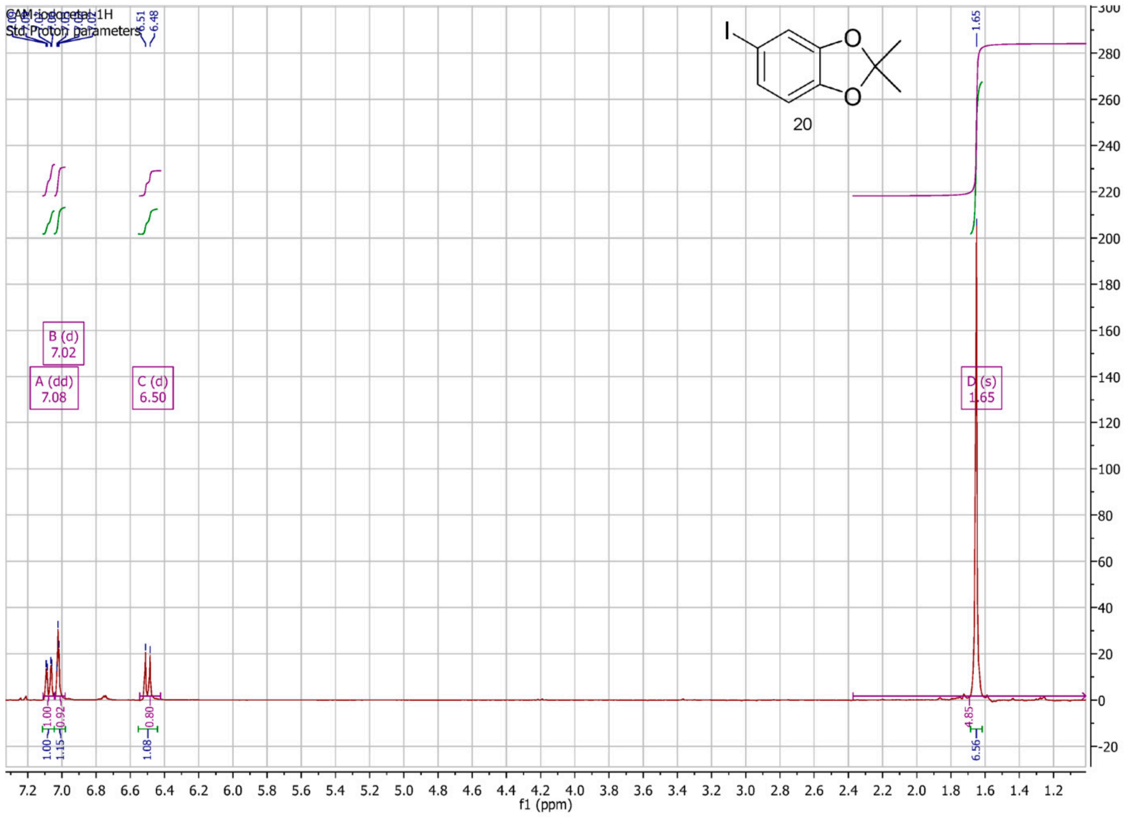

Figure S1. ^{1}H Spectra for 5-iodo-2,2-dimethylbenzo[*d*][1,3]dioxole (20).

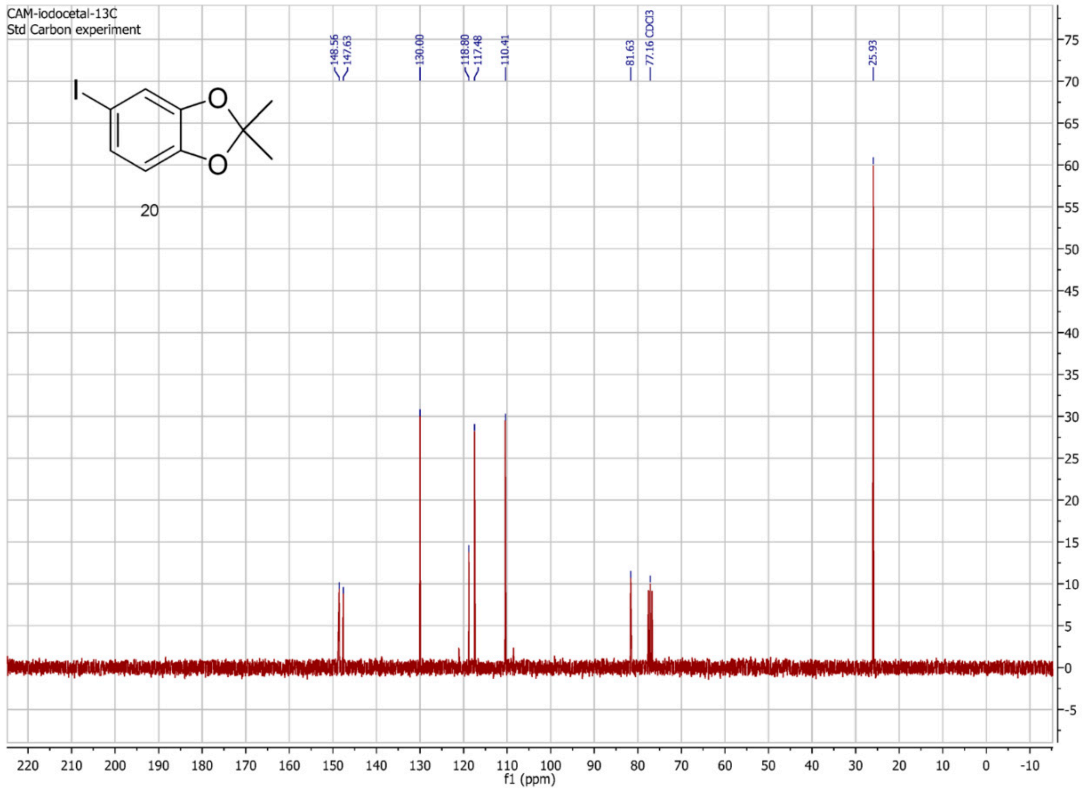

Figure S2. ^{13}C spectra for 5-iodo-2,2-dimethylbenzo[*d*][1,3]dioxole (20).

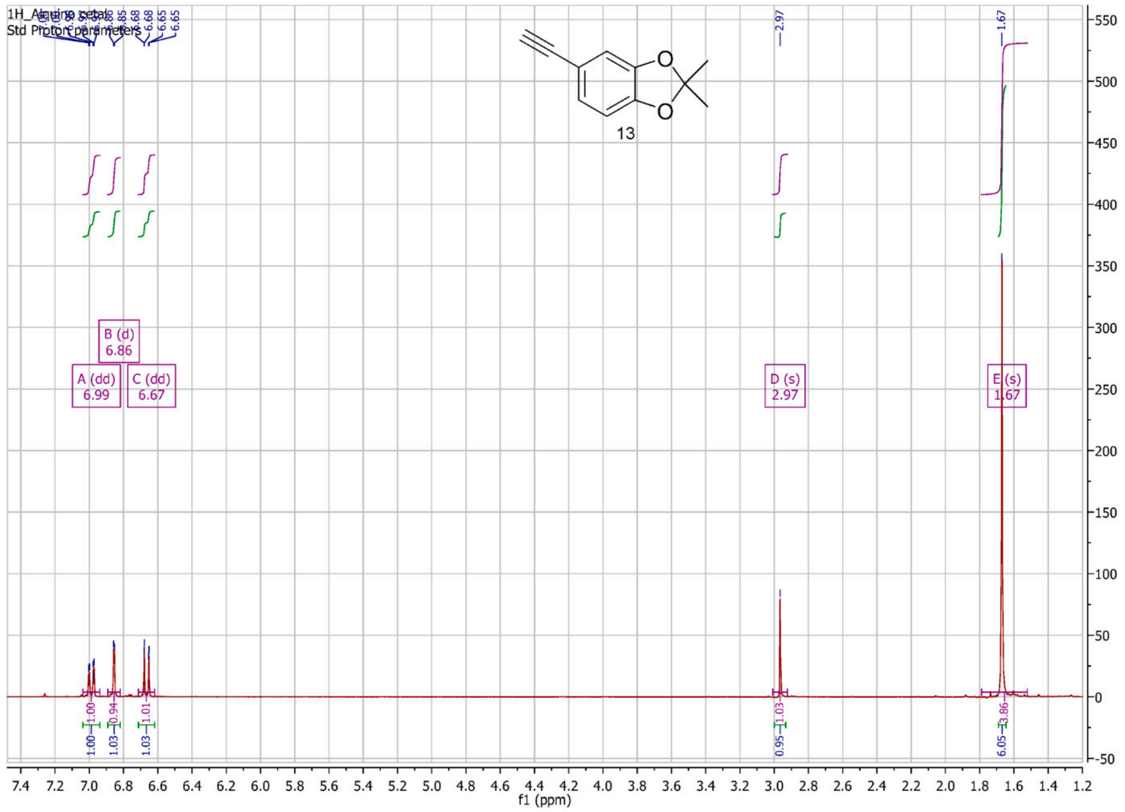

Figure S3. ^{1}H spectra for 5-ethynyl-2,2-dimethylbenzo[*d*][1,3]dioxole (13).

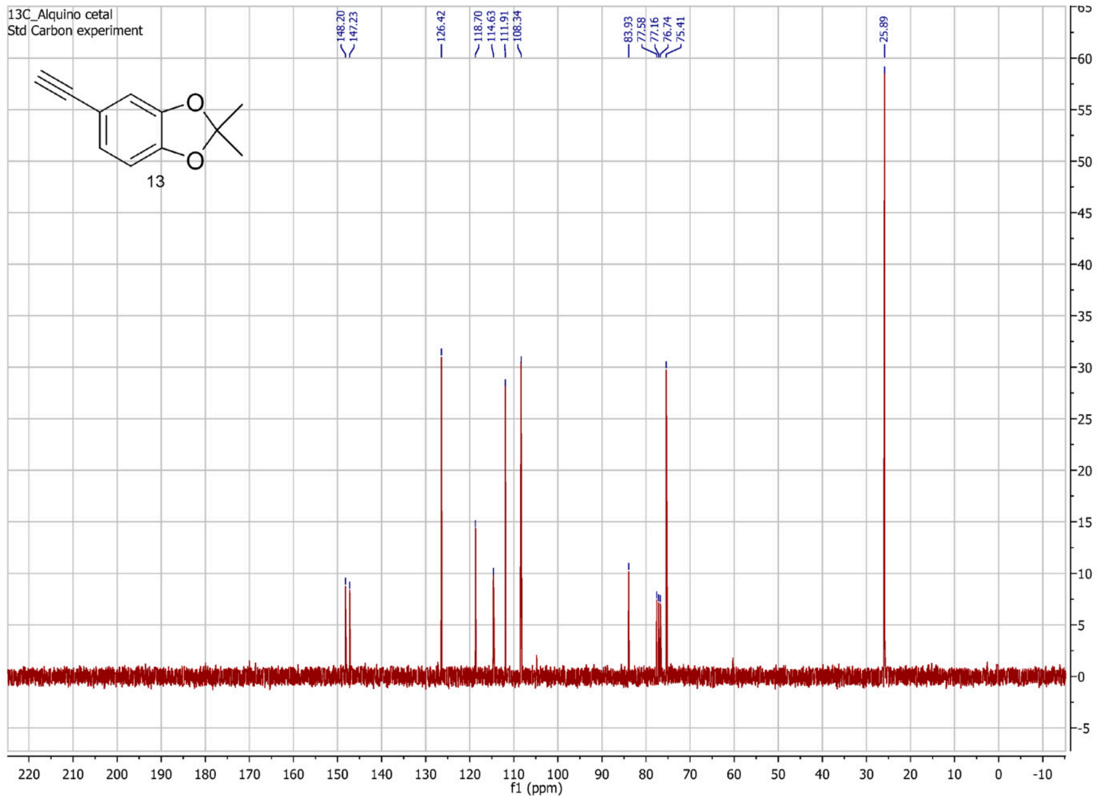

Figure S4. ^{13}C spectra for 5-ethynyl-2,2-dimethylbenzo[*d*][1,3]dioxole (13).

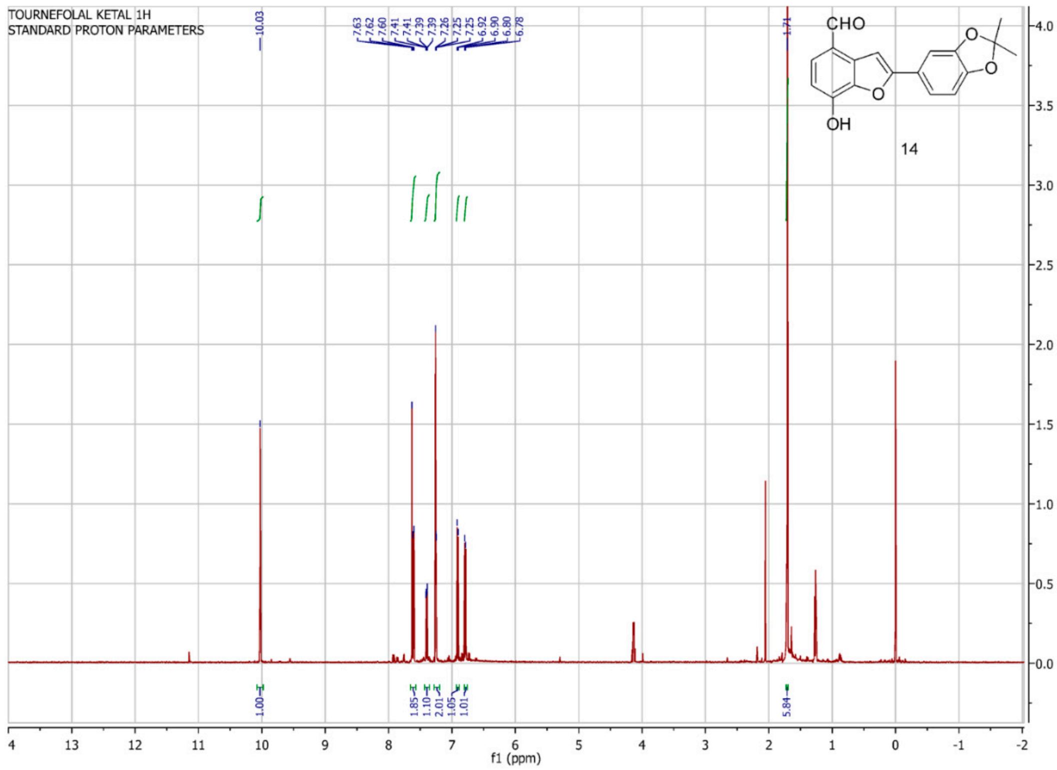

Figure S5. ^{1}H spectra for 2-(2,2-dimethylbenzo[*d*][1,3]dioxol-5-yl)-7-hydroxybenzofuran-4-carbaldehyde (14).

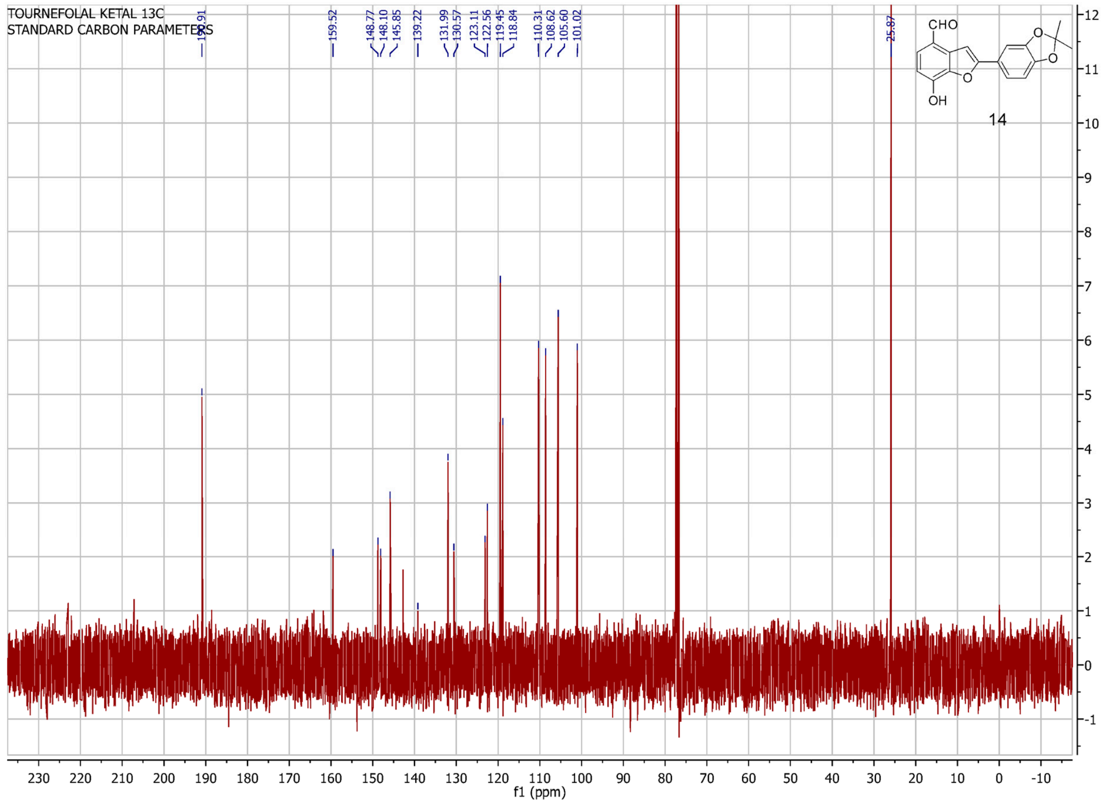

Figure S6. ^{13}C spectra for 2-(2,2-dimethylbenzo[*d*][1,3]dioxol-5-yl)-7-hydroxybenzofuran-4-carbaldehyde (14).

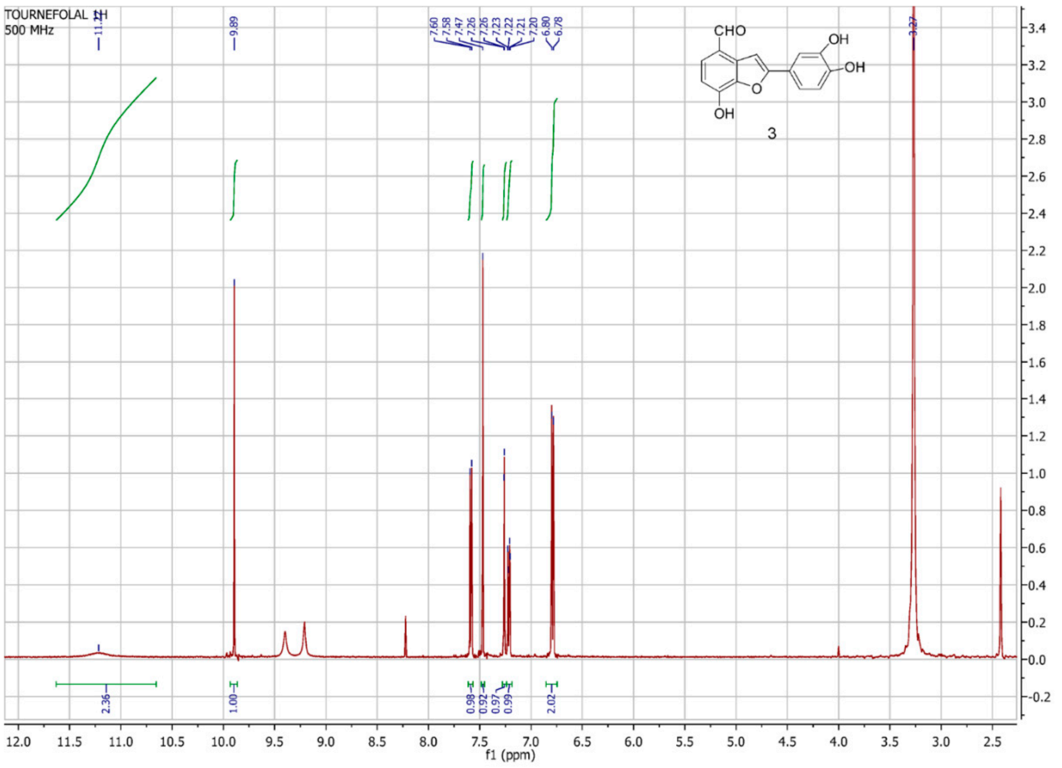

Figure S7. ^{1}H spectra for 2-(3,4-dihydroxyphenyl)-7-hydroxybenzofuran-4-carbaldehyde (Tournefolal) 3.

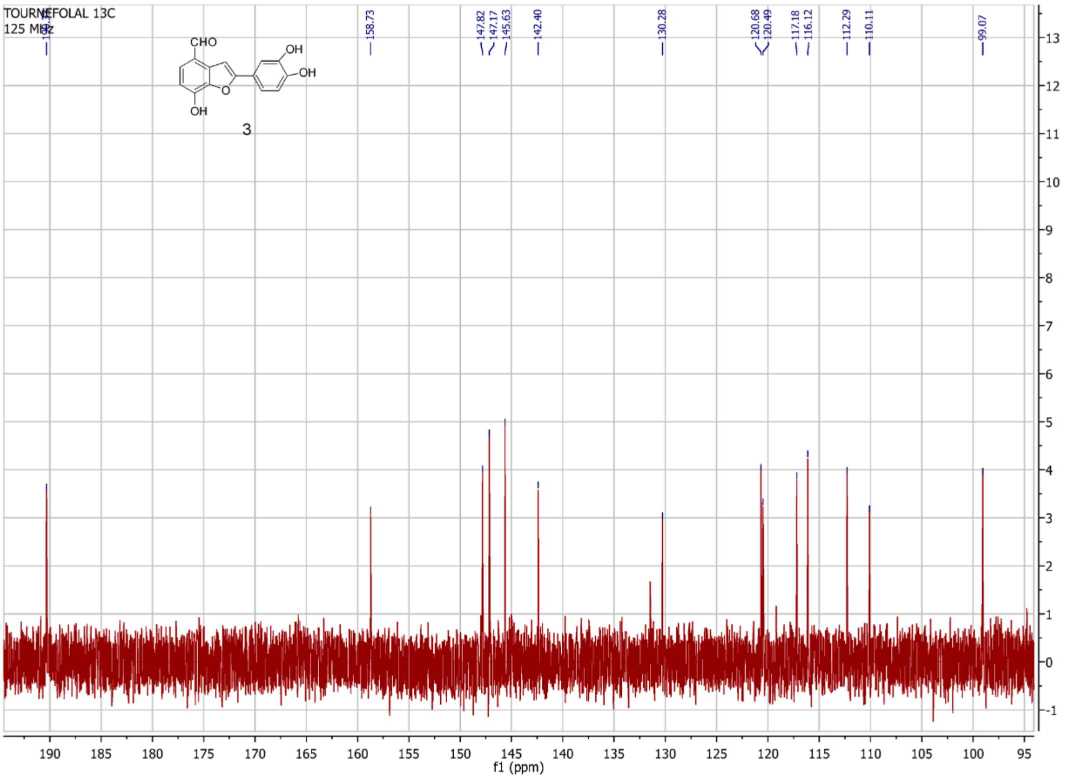

Figure S8. ^{13}C spectra for 2-(3,4-dihydroxyphenyl)-7-hydroxybenzofuran-4-carbaldehyde (Tournefolal) (3).

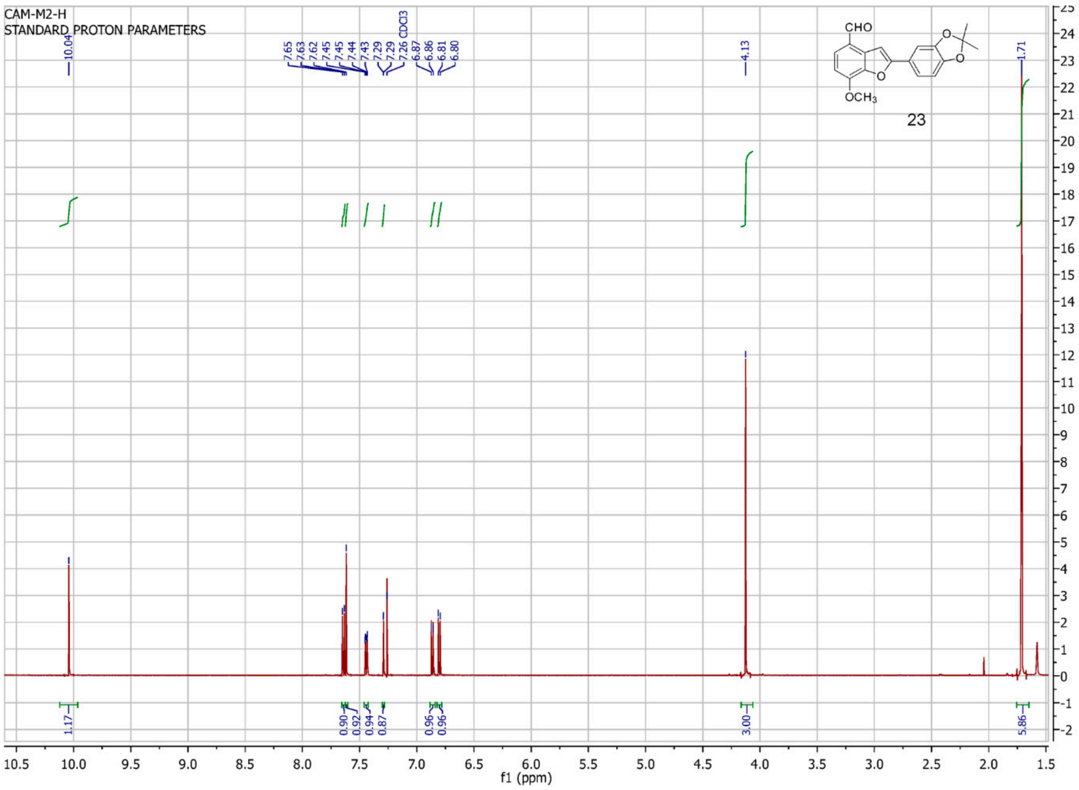

Figure S9. ^{1}H spectra for 2-(2,2-dimethylbenzo[*d*][1,3]dioxol-5-yl)-7-methoxybenzofuran-4-carbaldehyde (23).

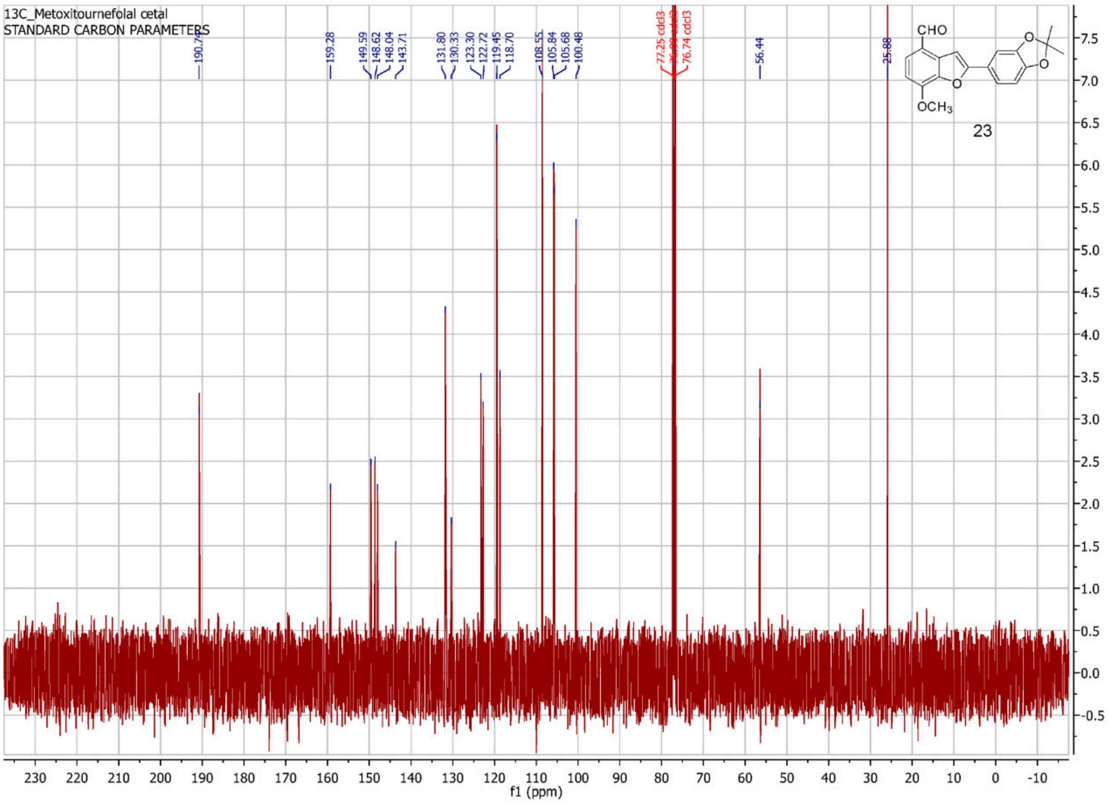

Figure S10. ^{13}C spectra for 2-(2,2-dimethylbenzo[*d*][1,3]dioxol-5-yl)-7-methoxybenzofuran-4-carbaldehyde (23).

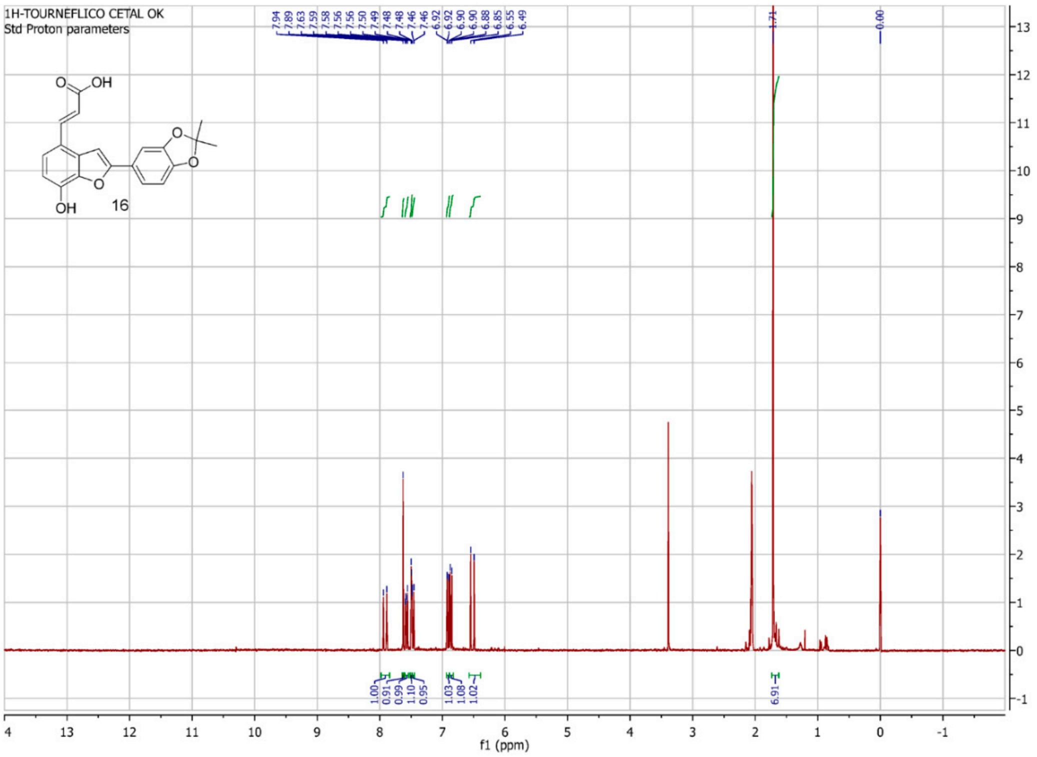

Figure S11. ^{1}H spectra (*E*)-3-(2-(2,2-dimethylbenzo[*d*][1,3]dioxol-5-yl)-7-hydroxybenzofuran-4-yl)acrylic acid (16).

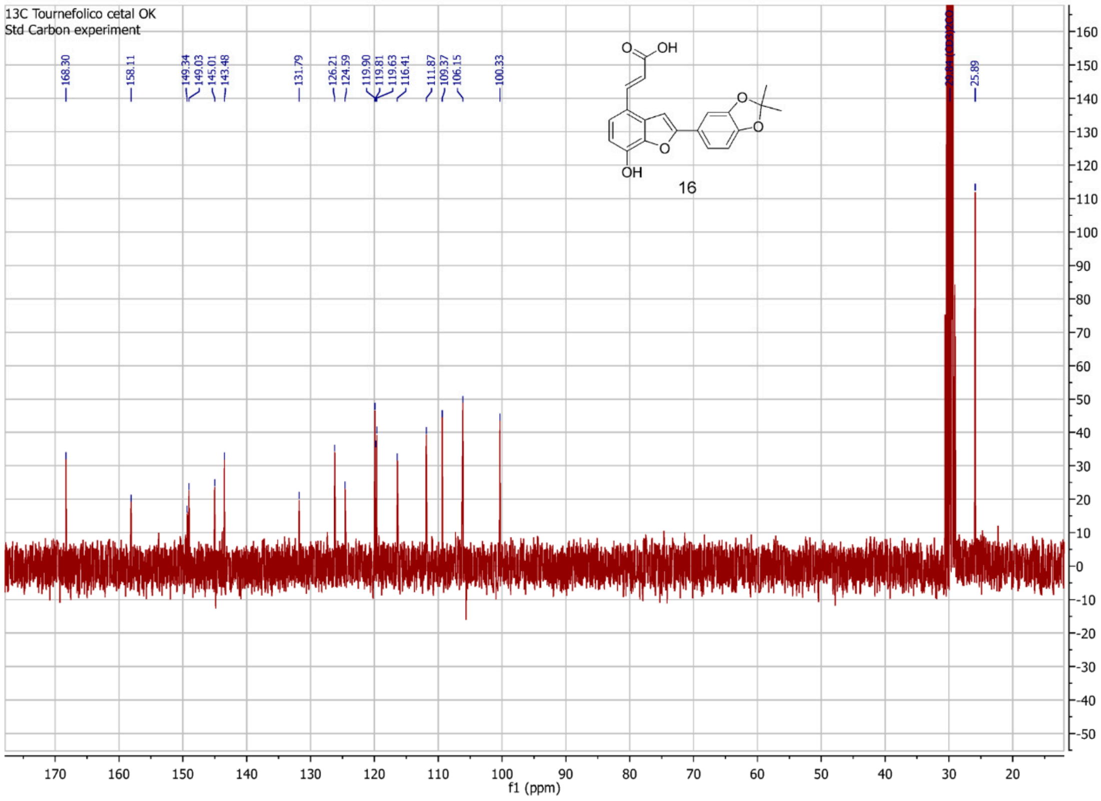

Figure S12. ^{13}C spectra for (*E*)-3-(2-(2,2-dimethylbenzo[*d*][1,3]dioxol-5-yl)-7-hydroxybenzofuran-4-yl)acrylic acid (16).

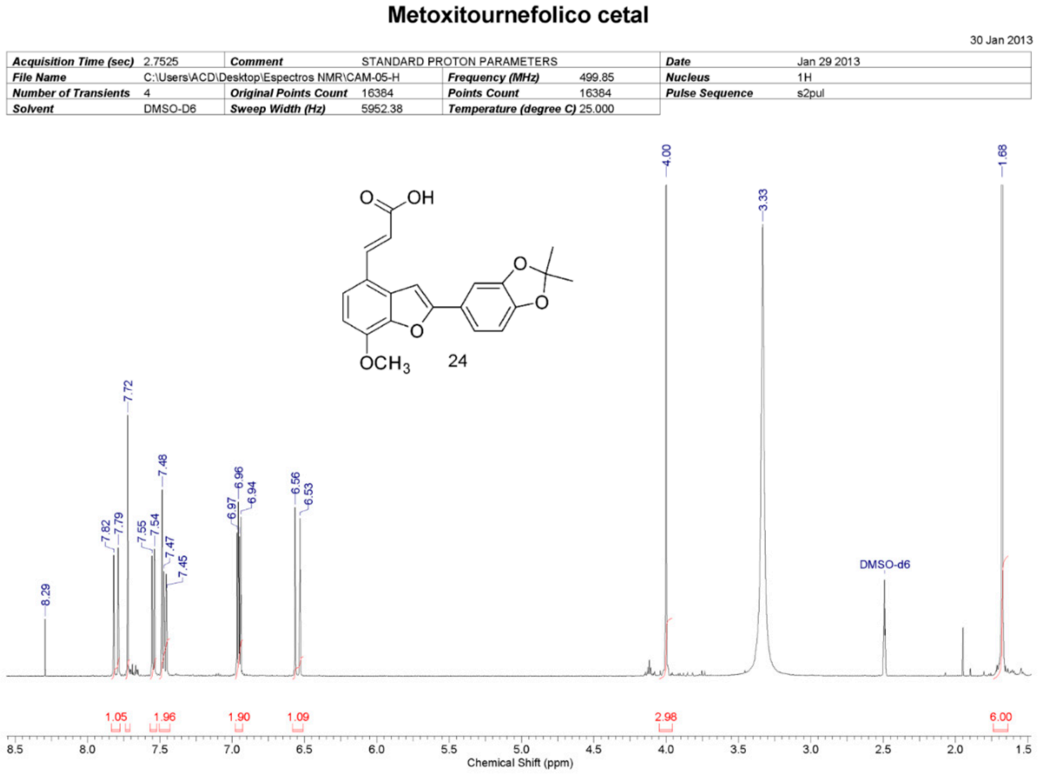

Figure S13. ^{1}H NMR spectra for (*E*)-3-(2-(2,2-dimethylbenzo[*d*][1,3]dioxol-5-yl)-7-methoxybenzofuran-4-yl)acrylic acid (24).

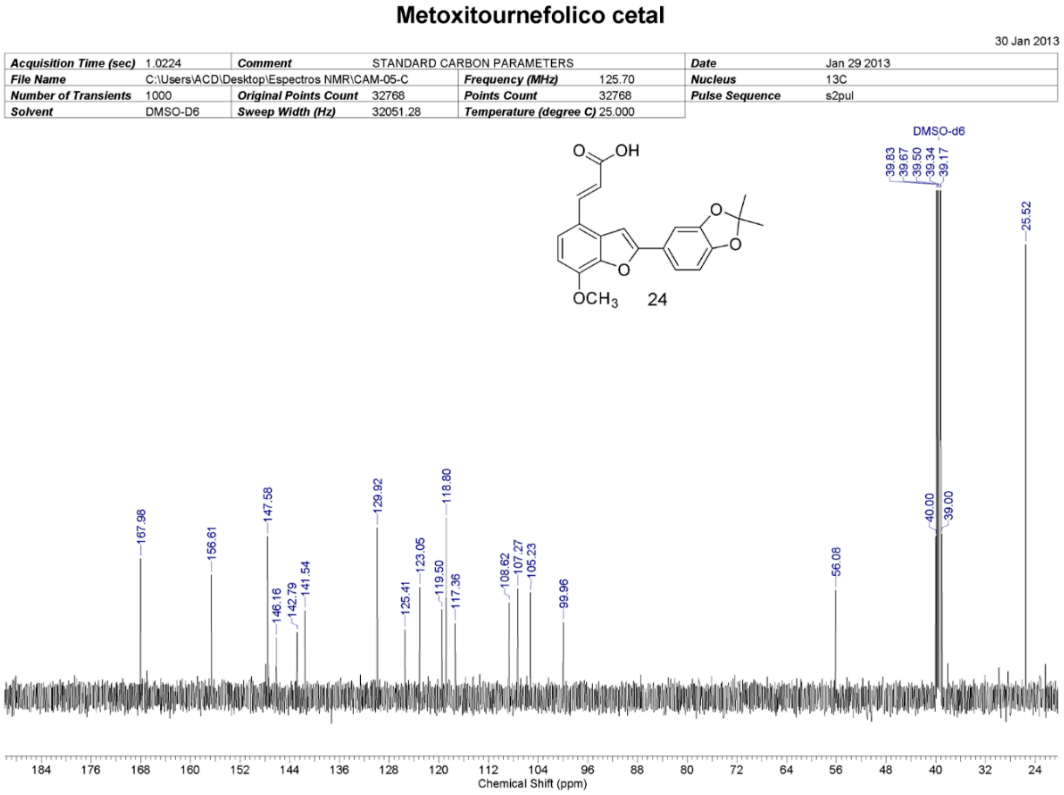

Figure S14. ^{13}C NMR spectra for (*E*)-3-(2-(2,2-dimethylbenzo[*d*][1,3]dioxol-5-yl)-7-methoxybenzofuran-4-yl)acrylic acid (24).
